# Safety and feasibility of contained uterine morcellation in women undergoing laparoscopic hysterectomy

**DOI:** 10.1186/s40661-018-0065-1

**Published:** 2018-10-30

**Authors:** Sarah Dotson, Alejandro Landa, Jessie Ehrisman, Angeles Alvarez Secord

**Affiliations:** 1 Department of Obstetrics and Gynecology, West Virginia University, 1 Medical Center Dr. PO Box 9186, HSC 4th floor, Morgantown, WV 26501-9186 USA; 2 Department of Obstetrics and Gynecology, Duke University Medical Center, Durham, NC 27710 USA; 3 Division of Gynecology Oncology, Department of Obstetrics and Gynecology, Duke Cancer Institute, Duke University Medical Center, Durham, NC 27710 USA

**Keywords:** Leiomyoma, Malignancy, Morcellation, Total laparoscopic hysterectomy

## Abstract

**Background:**

Widespread concerns have been raised regarding the safety of power morcellation of uterine specimens because of the potential to disseminate occult malignancy. We sought to assess the safety and feasibility of contained manual uterine morcellation within a plastic specimen bag among women with uterine neoplasms.

**Methods:**

A retrospective single-institution descriptive cohort study was conducted from 2003 to 2014. Patients with leiomyoma and/or uterine malignancy who underwent minimally invasive surgery with contained uterine manual morcellation were identified from surgical logs. Demographic data, pathology results, operative details and adjuvant treatments were abstracted.

**Results:**

Eighty-eight patients were identified; 35 with leiomyoma and 53 with endometrial cancer. The mean age was 48 and 60, respectively. Uterine size/weight was greater in women with leiomyoma compared to those with cancer (15.1 weeks/448 g vs. 10.7 weeks/322 g). Mean operative time was 206 min (range 115–391) for leiomyoma cases and 238 min (range 131–399) for cancer cases. Median length of stay was 1 day (range 0–3 days). There were no cases of occult leiomyosarcoma and all specimens were successfully manually morcellated within a bag. There were no intraoperative complications. Thirty-day postoperative complications occurred in 7 patients, including one readmission for grade (G) 1 vaginal cuff separation after intercourse, G1 port-site hematoma (1), G2 port-site cellulitis (1), G2 vaginal cuff cellulitis (2), G2 bladder infection (2), G2 pulmonary edema (1), and G1 musculoskeletal injury (1).

**Conclusions:**

Contained uterine hand morcellation is a feasible procedure with low peri-operative complication rates that allows for minimally invasive surgical procedures for women with large uterine neoplasms. Further evaluation is needed to assess survival outcomes for uterine malignancies.

## Background

Minimally invasive surgery (MIS) for hysterectomy provides patients with an alternative to laparotomy, particularly for uteri enlarged by leiomyomas or malignancy that are not amenable to vaginal hysterectomy. MIS can be performed either through conventional laparoscopic approaches or by utilizing the robotic platform. Advantages of MIS include faster return to normal activities, better cosmesis, decreased length of hospital stay, less blood loss, lower rates of infection and wound complications, less pain, and lower incidence of venous thromboembolism compared to laparotomy [1]. These benefits have been reported in women undergoing hysterectomy for both benign [1] and malignant conditions [2]. MIS for enlarged uteri presents gynecologic surgeons with the challenge of removing the uterus from the abdomen. Options for removal of the uterus traditionally include the following: laparotomy or “mini-laparotomy,” removal through the vagina with or without morcellation of the specimen, and removal via 10–15 mm laparoscopic port sites with intracorporeal morcellation, including the use of a power morcellator.

Concerns about the dissemination of undiagnosed uterine cancer using power morcellation have been raised. The FDA cautioned use of power morcellators in April 2014 after a patient undergoing hysterectomy for presumed leiomyoma was found to have leiomyosarcoma, disseminated throughout the abdomen by use of a power morcellator [3]. In November 2014, the FDA released a safety communication defining contraindications to power uterine morcellation: 1) removal of suspected fibroid tissue in peri- and postmenopausal women who are candidates for en-bloc resection (vaginal or via mini-laparotomy), and 2) removal of tissue known or suspected to contain malignancy [4]. The prevalence of occult uterine malignancy in patients with suspected leiomyoma is not known exactly, but multiple retrospective studies have estimated rates of malignancy at 0.2–1% in women undergoing uterine morcellation [5] compared to 0.23–0.49% in non-morcellated specimens [6]. Despite the low probability of malignancy, the risk of tumor dissemination has caused significant concerns regarding power morcellation, a moratorium of the procedure at select centers, and removal of some morcellation devices from the market.

In response to concerns over dissemination of uterine pathology, contained uterine morcellation within a specimen bag has been suggested as an approach to maintain a minimally invasive approach to hysterectomy [5–7]. However, the FDA warns that there are no studies examining the efficacy of contained “bag” morcellation. At our institution, we have used the technique of contained uterine manual (hand) morcellation within a specimen bag for more than a decade. The purpose of the study was to investigate the feasibility and safety of contained uterine morcellation. Our primary objectives were to describe intraoperative and postoperative complications, to determine the frequency of occult uterine leiomyosarcoma among patients with suspected leiomyoma, and to assess cancer outcomes among patients with known uterine malignancy following laparoscopic hysterectomy with contained “bag” morcellation.

## Methods

A retrospective single-institution descriptive cohort study was performed using chart review of cases from January 1, 2003 to December 31, 2014. The study was approved by the Duke University Institutional Review Board. Patients who underwent laparoscopic hysterectomy with contained uterine manual morcellation within a specimen bag were identified using chart review. Eligible study subjects were identified through a large institutional database of patients with endometrial cancer, as well as through review of surgical case logs maintained by the Division of Gynecologic Oncology for quality assurance purposes. Not all case logs were available for review and therefore this cohort may not include every eligible case during the study period. Two authors (SD and AL) reviewed available charts of patients who underwent laparoscopic hysterectomy to identify cases which included specimen morcellation within a bag. Patients with a post-operative diagnosis of endometrial cancer or uterine fibroids were included. All cases were performed by one of five Gynecologic Oncologists at our institution. While the study was not restricted to a particular type of specimen bag, the most commonly used technique at our institution is to place a 15 mm Endo Catch™ specimen retrieval bag through the vagina with a vaginal occlusion balloon, place the specimen in the bag under direct visualization and pull the opening of the bag through the vagina to expose the specimen. Morcellation was performed by hand using scissors or a scalpel to remove the specimen in small pieces that fit through the vagina. A similar technique is used via extended laparoscopic port sites. The decision to morcellate the uterus within a specimen bag was made intraoperatively when the uterus was too large to fit through the vagina intact, but the surgeon felt it could be safely removed using contained morcellation.

The two groups are described as separate cohorts, given two distinct patient populations for which uterine morcellation may be needed during minimally invasive hysterectomy. Data was abstracted by one of the authors (SD). Demographic data, pathology results, operative details, adjuvant treatments, disease recurrence and most recent disease status were abstracted. All data is descriptive for the two cohorts.

## Results

### Patient characteristics and operative findings

Eighty-eight patients were identified from retrospective chart review: 35 with leiomyoma and 53 with endometrial cancer. Baseline characteristics for the two groups are shown in Table 1. The mean age for patients with leiomyoma and uterine cancer was 48 and 60, respectively. The mean uterine size estimate on pre-operative exam was 15.1 and 10.7 weeks for women with leiomyoma and uterine cancer, respectively. Additional preoperative diagnoses are outlined in Table 1.

**Table 1:** Baseline characteristics

Characteristic	Leiomyoma (*N* = 35)	Uterine Cancer (*N* = 53)
Age	48 ± 6.5 [range 36–60]	60.1 ± 9.9 [range 38–81]
Race/ethnicity		
Caucasian	27 (77.1)	29 (54.7)
African American	5 (14.3)	23 (43.4)
Asian	0 (0)	1 (1.9)
Hispanic	2 (5.7)	0 (0)
Other	1 (2.9)	0 (0)
BMI	32.8 ± 9.2 [range 19.7–56.3]	37.5 ± 11.0 [range 19.2–64.5]
Parity^a^	1.5 ± 1.0 [range 0–3]	1.9 ± 1.8 [range 0–7]
Pre-op uterine size estimate (weeks)^b^	15.1 ± 2.7 [range 10–20]	10.7 ± 2.7 [range 6–14]
Menopausal	7 (20)	40 (75.5)
Prior Abdominal Surgery	23 (65.7)	27 (50.9)
Pre-op Diagnosis		
Leiomyoma	33 (94.3)	6 (11.3)
Endometrial cancer	0	50 (94.3)
Ovarian cyst/mass	10 (30.3)	2 (3.8)
Other^c^	10 (30.3)	7 (13.2)

Data are n(%) or mean ± SD unless otherwise specified. Data are presented as two separate cohorts given different disease states

^a^Missing data: *n* = 34 for leiomyoma group

^b^Missing data: *n* = 21 for leiomyoma group, *n* = 17 for uterine cancer group

^c^Includes endometrial intraepithelial neoplasia (3), abnormal uterine bleeding (4), family history of cancer (1), terminal ileum cancer (1), anemia (1), breast cancer (1), pelvic pain (1) and history of rectovaginal fistula repair (1) in the leiomyoma group and postmenopausal bleeding (2), atypical glandular cells (2), cirrhosis (1), cervical stenosis (2), chronic kidney disease (1), ventral hernia (1), atypical spindle cells (1) and pelvic organ prolapse (1) for the endometrial cancer group

Intraoperative and postoperative findings are shown in Table 2. The majority of cases were performed using a robotic platform. Mean operative time was 206 min (range 115–391) for leiomyoma cases and 238 min (range 131–399) for cancer cases. The mean uterine weight was 448 g (140 g - 1076 g) in women with leiomyoma and 322 g (135 g - 611 g) in those with malignancy. Length of inpatient hospital stay was 1 day, range 0–3 days) in both groups. The most frequent method for uterine specimen morcellation was contained manual morcellation within a specimen bag inside the vagina (82.9% for leiomyoma and 98.1% for uterine cancer). Alternative methods used included contained manual morcellation through an extended laparoscopic port site and contained vaginal morcellation of a fibroid separate from the uterus. In one case, the cervix and lower portion of the uterus were morcellated within a bag in the vagina, but the remainder of the specimen was removed via mini-laparotomy, still contained within the specimen bag. No cases of occult leiomyosarcoma were identified. There were no intraoperative complications in either group. One patient received planned transfusion of fresh frozen plasma to correct INR secondary to known coagulopathy from cirrhosis (estimated blood loss 100 mL). Thirty-day postoperative complications occurred in 7 patients, including one readmission for grade (G) 1 vaginal cuff separation after intercourse, as well as G1 port-site hematoma (1), G2 port-site cellulitis (1), G2 vaginal cuff cellulitis (2), G2 bladder infection (2), G2 pulmonary edema (1), and G1 musculoskeletal injury (1). Two patients were suspected of having small vaginal cuff hematomas, but no imaging was obtained for confirmation, and symptoms resolved without intervention. Mean length of follow-up was 24 (range 1–85) months for patients with uterine cancer and 31 (range 3–89) months for patients with leiomyoma.

**Table 2:** Operative and postoperative characteristics

Perioperative characteristics	Leiomyoma (*N* = 35)	Uterine cancer (*N* = 53)
Robotic platform	26 (74.3)	34 (64.2)
Uterine weight (grams)^a^	448.35 ± 194.9 [range 140–1076]	321.77 ± 102.1 [range 135–611]
Intra-op size estimate (weeks)^b^	14.8 ± 2.6 [range 10–20]	12.5 ± 2.2 [range 6–16]
Operative time (minutes)^c^	206.0 ± 76.3 [range 115–391]	237.5 ± 67.0 [range 131–399]
Abdominal/pelvic Adhesions	13 (37.1)	35 (34.0)
Length of stay^d^	0.97 ± 0.5 [range 0–3]	1.12 ± 0.4 [range 0–3]
Mode of morcellation		
Port site	3 (8.6)	0 (0)
Vaginal	29 (82.9)	52 (98.1)
Other	3 (8.6)	1 (1.9)
Post-op Diagnosis		
Leiomyoma	35 (100)	40 (75.5)
Endometrial cancer^e^	1 (2.9)	53 (100)
Benign ovarian neoplasm	9 (25.7)	6 (11.3)
Adenomyosis	13 (37.1)	18 (33.9)
Pelvic adhesive disease	8 (22.9)	9 (17.0)
Other^f^	18 (51.4)	12 (22.6)
Post-op complications^g^	3 (8.6)	4 (7.5)
Length of follow-up (months)	31 ± 21 [range 3–89]	24 ± 21 [range 1–85]

Data are n (%) or mean ± SD unless otherwise specified

^a^Missing data: *n* = 34 for leiomyoma group, *n* = 52 for uterine cancer group

^b^Missing data: *n* = 30 for leiomyoma group, *n* = 24 for uterine cancer group

^c^Missing data: *n* = 33 for leiomyoma group, *n* = 52 for uterine cancer group

^d^Missing data: *n* = 52 for uterine cancer group

^e^Incidental finding of Stage IA, grade 1 endometrioid adenocarcinoma, no adjuvant therapy

^f^Includes endometrial intraepithelial neoplasia (3), abnormal uterine bleeding (4), family history of cancer (1), terminal ileum cancer (1), anemia (1), breast cancer (1), pelvic pain (1), abdominal wall mesothelioma (1), retroperitoneal fibrosis (2), urethral diverticulum (1) ovarian cancer (1), endometrial polyp (3), endometriosis (3)and history of rectovaginal fistula repair (1) in the leiomyoma group and hydrosalpinx (1), endometriosis (1), ventral hernia (1) and appendicitis (1) for the endometrial cancer group

^g^Includes readmission for grade (G) 1 vaginal cuff separation after intercourse, as well as G1 port-site hematoma (1), G2 port-site cellulitis (1), G2 vaginal cuff cellulitis (2), G2 bladder infection (2), G2 pulmonary edema (1), and G1 musculoskeletal injury (1)

### Uterine malignancies

Fifty-three patients underwent hysterectomy for uterine cancer and the histologic subtypes were as follows: 75.5% endometrioid, 9.4% serous and 1.9% clear cell adenocarcinomas, and 13.2% carcinosarcoma (Table 3). One case of grade 1 endometrioid adenocarcinoma contained sections with grade 3 signet ring cell differentiation. Among the 40 cases of endometrioid adenocarcinoma, 55% were grade 1, 42.5% grade 2 and 7.5% grade 3. Cytologic washings were positive in 5 cases (9.4%), suspicious or indeterminate in 6 cases (11.3%), and not collected in one case. Spillage of tumor was described in 2 cases, which occurred during hysterectomy and prior to placing the specimen in the plastic bag. One of these patients died of recurrent IB carcinosarcoma within 1 year, and the second patient (IB, grade 2 endometrioid adenocarcinoma) was offered and declined radiation therapy, and was alive at 2.5 months following surgery with no evidence of disease. Distribution of cancer stage is shown in Table 3.

**Table 3:** Uterine cancer characteristics, adjuvant therapy and disease status

Characteristic	Uterine cancer (*N* = 53)
Pre-op Histology (*n* = 49)	
Endometrioid	34 (69.4)
Serous	6 (12.2)
Clear cell	3 (6.1)
Carcinosarcoma	3 (6.1)
Other/unspecified	7 (14.3)
Post-op Histology	
Endometrioid	40 (75.5)
Serous	5 (9.4)
Clear cell	1 (1.9)
Carcinosarcoma	7 (13.2)
Grade	
1	21 (39.6)
2	15 (28.3)
3	17 (32.1)
Stage	
IA	28 (52.8)
IB^a^	15 (28.3)
II	1 (1.9)
IIIA	2 (3.8)
IIIC1	3 (5.7)
IIIC2	2 (3.8)
IVB	2 (3.8)
Lymph nodes	
Positive	6 (11.32)
Negative	29 (54.7)
Not collected	18 (34.0)
Pelvic Washings	
Positive	5 (9.4)
Negative	41 (77.4)
Indeterminate/suspicious	6 (11.3)
Not collected	1 (1.9)
Adjuvant Therapy^b^	
Radiation	13 (24.5)
Chemotherapy	10 (18.9)
Hormonal	4 (7.6)
Recurrence rate	7 (13.2)
Median time to recurrence (days)	395 [range 128–539]
Status of disease	
No evidence of disease	43 (81.1)
Alive with disease^c^	3 (5.7)
Died of disease	5 (9.4)
Died of intercurrent disease	1 (1.9)
Died of unknown cause^d^	1 (1.9)

Data are n(%) unless otherwise specified

^a^Depth of invasion could not be determined in one case and is included in the Stage 1B group

^b^Chemotherapy: Carboplatin/Taxol (6), Carboplatin/Taxol + Cisplatin (with radiation) (1), Carboplatin/Taxol + Doxil (1); Ifosfamide/Taxol (2). Radiation: vaginal brachytherapy (5), external beam radiation (4), IMRT (2), vaginal brachytherapy + external beam radiation (1). Hormonal: Megace (2), Megace + Tamoxifen (1), Megace + Anastrozole (1)

^c^(1) IIIC-2, grade 2 endometrioid, disease-free survival 7.8 months (2) IA, grade 2 endometrioid, disease-free survival 13.2 months (3) IB, grade 3 endometrioid, disease-free survival 4.3 months

^d^Most likely this patient died of disease given her disease distribution with distant metastases

Fifteen patients with uterine cancer received adjuvant treatment with chemotherapy or radiation, and four patients received hormonal therapy (Table 3). Median length of follow up was 546 days (range 34–2535 days). There were 7 deaths in the uterine cancer group (13.2%), and 5 were attributable to the patient’s cancer (9.4%) (Table 4). Patients who died of their cancer had the following disease stages, histologic subtypes and adjuvant therapies: 1B serous adenocarcinoma who received adjuvant pelvic radiation and died with disseminated disease in the setting of end-stage renal disease; recurrent IA, grade 2 endometrioid who received no adjuvant therapy; IVB carcinosarcoma who declined adjuvant therapy; IIIC-2 and IIIA serous adenocarcinoma who each received adjuvant chemotherapy and pelvic radiation. One patient with Stage IA, grade 1 endometrioid adenocarcinoma died of severe pulmonary disease unrelated to malignancy 10 months after surgery. One patient with Stage IIIC-1 endometrioid adenocarcinoma died of unknown cause, but in the setting of recurrent, progressive disease for 1 year. Mean survival time among patients whose death was attributed to their uterine malignancy was 342 days (range 84–549 days).

**Table 4:** Clinicopathologic characteristics, adjuvant therapy, and survival outcomes of the deceased

Stage, grade, histology	Uterine size (weeks)	Adjuvant therapy	Site of recurrence	Progression free survival (months)	Overall survival (months)	Death status
IA, 1 endometrioid	14	none	N/A	N/A	51.2	DICD (pulmonary disease)
IA, 2 endometrioid	14	none	carcinomatosis	16.1	18.3	DOD
IB carcinosarcoma	12	WPRT	RLQ mass	8.3	10.7	DOD
IIIA serous	Not recorded	Carboplatin/ Taxol IMRT	carcinomatosis	14.1	17.4	DOD
IIIC-1, 3 endometrioid	13	Carboplatin/ Taxol	Pulmonary, mediastinal	18.0	30.6	Unknown cause^a^
IIIC-2 serous	15	Carboplatin/ Taxol External beam pelvic radiation	N/A	N/A	7.7	DOD
IVB carcinosarcoma	10	none	carcinomatosis	1.6	2.8	DOD

*WPRT* whole pelvic radiation therapy, *IMRT* intensity-modulate radiation therapy, *DOD* died of disease, *DICD* died of intercurrent disease

^a^Most likely this patient died of disease given her disease distribution with distant metastases

## Discussion

Determining alternative and safe procedural techniques for uterine morcellation and specimen extraction are necessary to permit MIS for women with large uteri, not amenable to vaginal hysterectomy. MIS has several advantages over laparotomy, including better surgical outcomes and quality of life in the majority of patients [1, 8]. Siedoff et al. published a decision analysis comparing surgical risks of laparotomy with risks of disseminated occult malignancy during laparoscopy for women with large leiomyoma, and predicted that laparoscopic hysterectomy was associated with slightly better 5-year overall survival and improved quality-adjusted life years [9]. Thus, on a population level, the benefits of MIS for the large majority of patients may outweigh the very small risk of disseminating occult malignancy in very few patients. This brings into consideration the key medical principle of “primum non nocere” or non-maleficence. The conflict regarding power morcellation highlights the juxtaposition of utilitarianism and non-maleficence, emphasizing the need to develop procedural techniques to provide safe MIS options for women with enlarged uteri.

Our cohort of patients with leiomyoma and uterine malignancy represents the largest published series of patients undergoing contained uterine morcellation to date. Contained uterine hand morcellation within a specimen bag appears to be a feasible technique based on our experience with a large group of patients undergoing minimally invasive hysterectomy for both benign and malignant uterine neoplasms. Our patient population was diverse with respect to uterine size, body mass index, previous abdominal surgeries and pelvic adhesions, and safe removal of the uterus was achieved with no intraoperative complications. The technique uses relatively low cost specimen bags and does not require additional advanced surgical skills. Furthermore, for patients with uterine cancer who require adjuvant therapy, minimally invasive surgery allows for more rapid recovery with fewer wound complications leading to fewer delays in starting subsequent therapy. Further evaluation is needed to support our findings, particularly with respect to uterine cancer outcomes.

Multiple other studies have used similar techniques for contained uterine morcellation for both benign [10, 11] and malignant uterine neoplasms [12, 13]. Cohen et al. described power morcellation through a laparoscopic port site within a specimen bag for patients with leiomyoma (*n* = 73), demonstrating efficiency in operative time and no complications with a wide range of uterine sizes [10]. Favero et al. and Montella et al. have published small prospective case series (*n* = 30, 12 respectively) of patients with uterine cancer who underwent contained uterine hand morcellation through the vagina, both demonstrating the feasibility of the technique [12, 13]. Serur et al. described contained hand morcellation via an extended laparoscopic port site for patients with benign pathology [11]. Together, these studies and our findings indicate that contained uterine morcellation is feasible with low rates of complications and conversion to laparotomy.

Different types of specimen bags were used in each study, demonstrating that specific specialty equipment is not required for this technique. Currently there are three FDA approved tissue retrieval devices (Applied Medical Specimen Retrieval System and Tissue Containment System (Rancho Santa Margarita, CA) and Cook LapSac Tissue Entrapment Pouch (Mudellein, IL)) [14]. These devices are approved to contain and isolate tissue during, or prior to, surgical removal and/or extracorporeal manual morcellation, but are contraindicated for use with powered cutting devices (power morcellators, electrosurgical and laser instruments). Solima et al. performed a prospective evaluation of the Endo Catch™ bag in 12 patients and revealed no gross rupture during morcellation, but identified four minimal ruptures using diluted methylene blue. These findings demonstrate concern over bag integrity and a potential route for tumor dissemination. Our study was retrospective and no specific integrity testing was performed on the bags before or after morcellation so unnoticed small spillage of tissue could have occurred.

In our series, none of the women who underwent laparoscopic hysterectomy and contained manual uterine morcellation for leiomyoma had an occult malignancy. In addition, we had no reports of postoperative intra-abdominal dissemination of benign uterine pathology including leiomyomatosis and endometriosis which have been described with an estimated prevalence of 0.5–1.2% following mechanical morcellation [5, 15–17]. During the time period of the study we did not routinely conduct preoperative testing such as endometrial biopsies or MRIs to assess for malignancy. Recent research efforts have focused on preoperative risk stratification of patients with large leiomyoma considering minimally invasive surgery in order to correctly select appropriate candidates for laparoscopic hysterectomy. Known risk factors for uterine malignancy include older age, menopausal status, exposure to tamoxifen or radiation therapy and a history of certain hereditary cancer syndromes [18]. Past studies have shown that large uterine size [19, 20] and rapidly increasing uterine size [21] are not associated with an increased risk of uterine sarcoma. The International Society for Gynecologic Endoscopy (ISGE) identified black race, increasing age, ≥ 5 years tamoxifen use, hereditary leiomyomatosis and renal cell carcinoma (HLRCC), prior pelvic radiation, and personal history of childhood retinoblastoma as risk factors for sarcoma. The ISGE has published clear guidelines for preoperative evaluation and consent prior to hysterectomy for uterine leiomyoma. Using a retrospective cohort design, Ricci et al. demonstrated that young age is associated with very low risk of occult malignancy and no cases of leiomyosarcoma were identified among a cohort of reproductive age women [22]. A combined approach of preoperative risk stratification and contained uterine morcellation may allow gynecologic surgeons to offer a minimally-invasive approach to hysterectomy for the majority women with large uteri, while quelling fears of intra-abdominal dissemination of malignant tissue.

With regard to endometrial cancer, MIS became the standard surgical approach based on the initial experience reported by Walker et al. [2], which showed short-term advantages of laparoscopy compared to laparotomy, including shorter hospital stays, fewer perioperative complications, reduced blood loss, similar incidence of metastatic disease (17% of patients in both groups) and similar recurrence rates (11.4% in the laparoscopy group and 10.2% in the laparotomy group; hazard ratio for laparoscopy 1.14; 95% CI, 0.92 to 1.46) [23]. However, the study results were inconclusive because laparoscopy could not be demonstrated with 95% confidence to have a hazard ratio below the predetermined threshold of 1.4 for noninferiority [24]. While the study may have been underpowered to detect differences in survival between groups, particularly certain high-risk histologic subtypes, laparoscopy has become the dominant surgical approach for women with earlier stage endometrial cancer [23, 24]. Significant uterine enlargement that precluded a vaginal hysterectomy was an exclusion criterion for the LAP2 study. Therefore, the findings from LAP2 may not be generalizable to women with larger uteri or to the patients in our study who had enlarged uteri requiring morcellation. However, recent SEER data indicated that MIS hysterectomy was not associated with worse overall (HR, 0.89; 95% CI, 0.75–1.04) or cancer-specific (HR, 0.83; 95% CI, 0.59–1.16) mortality compared to abdominal hysterectomy [25].

Evaluation of survival outcomes in our study is difficult given that our sample size is small and heterogeneous, and follow-up time is limited. Overall five-year survival rates for women with endometrial cancer range widely based on stage of disease: stage IA, 88%; stage IB, 75%; stage II, 69%; stage IIIA, 58%; stage IIIB, 50%; stage IIIC, 47%; stage IVA, 17%; and stage IVB, 15% [26]. Many women with endometrial cancer who died in our study had advanced disease or aggressive histologic subtypes associated with worse survival (serous adenocarcinoma, carcinosarcoma, and grade 3 endometrioid adenocarcinoma). One patient with stage IA, grade 2 endometrioid carcinoma was diagnosed with carcinomatosis at the time of recurrence. Carcinomatosis is a pattern of recurrence that is concerning in the setting of morcellation. Intraperitoneal spillage was not reported during her initial surgery, but in general we suspect that gross spillage of tumor in the peritoneal cavity may worsen prognosis. While tumor spillage may occur during laparotomy, it may be more frequent during MIS where manipulation of the uterus through the vagina or into a bag for extraction is required. Montella et al. have reported short-term cancer outcomes for 12 endometrial cancer patients who underwent contained uterine morcellation [13]. All patients had grade 1 or 2 endometrioid adenocarcinoma. Nine patients had Stage IA, two had Stage 1B, and one had Stage IIIA disease. All were alive without evidence of recurrence at a median of 18 months of follow-up. Favero et al. had a median follow up of 20 months and reported 24-month survival of 74%. Four patients with metastatic nodal disease had died with distant metastasis [12]. In addition to concern of spreading malignant cells, morcellation raises new challenges in pathology interpretation of disrupted tissue specimens. [27] Pathologic evaluation of morcellated uteri is more challenging and there is a possibility that smaller uterine tumors were missed.

Strengths of this study include the relatively large sample size for describing a single surgical technique and the diversity of patients with respect to age, BMI, uterine size and pathology. Limitations include retrospective design, inability to identify all cases performed at the institution, lack of comparison group for outcomes, heterogeneity of tumor specimen retrieval bags and methodology for extraction, and short length of follow-up with respect to cancer-related outcomes. Additionally, hand morcellation can be a time-consuming process, particularly for large uteri. More research is needed to compare different techniques for specimen morcellation and extraction with respect to operative time and complications. The safety of power morcellation within a specimen bag should be further investigated as this particular technique has the potential to maximize efficiency while protecting against dissemination of tissue. Furthermore, the complexity of this technique may require more advanced training to ensure safety in the hands of novice users.

## Conclusions

Our initial results indicate that contained manual morcellation of uterine specimens is both feasible and safe from an intra-operative and immediate postoperative perspective for women with leiomyoma. Our study, however does not address the safety of the technique among the small number of women who are found to have leiomyosarcoma at the time of surgery. Further prospective research is needed to confirm the safety of FDA-approved specimen retrieval devices, to develop a systematic approach to preoperative risk stratification for patients considering MIS for large uterine neoplasms, and to assess this technique further in women with uterine malignancies to determine safety with respect to long-term oncologic outcomes.

## Abbreviations

FDA: Food and Drug Administration
MIS: Minimally invasive surgery
